# Physical therapy counts: counting physical therapists worldwide

**DOI:** 10.1186/1472-6963-14-S2-O23

**Published:** 2014-07-07

**Authors:** Catherine Sykes, Tracy Bury, Brenda Myers

**Affiliations:** 1 World Confederation for Physical Therapy, London, SW1V1RB, UK; 2 Faculty of Health Sciences, University of Sydney, Sydney, NSW 2006, Australia; 3 Faculty of Health Sciences, Trinity College Dublin, Dublin 2, Ireland

## Background

It is evident that if something is not counted, and physical therapists (PTs) are not generally counted in international health statistics, no one knows how many there are, neither can they measure their contribution to population health or monitor their migration. The World Health Report [1] provides statistics on doctors and nurses primarily. Few data are reported on other health personnel.

To address the burgeoning needs of populations living with non-communicable diseases, disability and older people every health professional will need to be involved. PTs with their expertise in maintaining and restoring people’s maximum movement and functional ability are well placed to take a key role. However with the paucity of information about the profession health, policy makers and planners are disadvantaged.

## Materials and methods

To fill the information gap, the World Confederation for Physical Therapy (WCPT) has developed an annual data collection which generates a country profile that includes information on the WCPT member organisations (MOs), the number of PTs, scope of practice, education and regulation.

WCPT MOs were asked for their top priority information requirements. Together with the priorities of WCPT this information provided the base for a set of questions. A sample of national health workforce surveys was reviewed for relevant data items. Where available, international standard classifications were used. A data advisory group involved people from all WCPT regions. Following feedback the data items were reviewed and pilot tested by representatives of the majority of the WCPT MOs. An online data capture tool was built and pilot tested. The first collection was made in 2012.

## Results

In 2012, 69 of 106 WCPT MOs provided data, a 65% response rate. The analysis enabled reporting on numbers of PTs who were members of WCPT MOs and an estimate of the number of PTs in the country of the responding MOs, which paired with population data from the World Bank provided an estimate of the PT to population ratio. The ratio varied between 0.002 PTs per 1000 population to 2.82 per 1000 population.

## Conclusions

The WCPT collection is the only global collection on PT workforce, education, regulation and practice. Whilst the collection is in its infancy and data quality and reporting requires improvement, it can provide information to governments, inter- and non-governmental agencies, the profession and the public. In time, longitudinal data may illustrate the changes in the country profiles as a response to health workforce migration, policies and planning.

